# Using DNA-based stable isotope probing to reveal novel propionate- and acetate-oxidizing bacteria in propionate-fed mesophilic anaerobic chemostats

**DOI:** 10.1038/s41598-019-53849-0

**Published:** 2019-11-22

**Authors:** Hui-Zhong Wang, Xiao-Meng Lv, Yue Yi, Dan Zheng, Min Gou, Yong Nie, Bing Hu, Masaru K. Nobu, Takashi Narihiro, Yue-Qin Tang

**Affiliations:** 1 College of Architecture and Environment, Sichuan University, No. 24, South Section 1, First Ring Road, Chengdu, Sichuan 610065 China; 2 Institute of New Energy and Low-Carbon Technology, Sichuan University, No. 24, South Section 1, First Ring Road, Chengdu, Sichuan 610065 China; 3 Department of Energy and Resources, College of Engineering, Peking University, Beijing, 100871 China; 4 Bioproduction Research Institute, National Institute of Advanced Industrial Science and Technology (AIST), Tsukuba, 305-8566 Japan

**Keywords:** Environmental biotechnology, Environmental microbiology

## Abstract

Propionate is one of the most important intermediates of anaerobic fermentation. Its oxidation performed by syntrophic propionate-oxidizing bacteria coupled with hydrogenotrophic methanogens is considered to be a rate-limiting step for methane production. However, the current understanding of SPOB is limited due to the difficulty of pure culture isolation. In the present study, two anaerobic chemostats fed with propionate as the sole carbon source were operated at different dilution rates (0.05 d^−1^ and 0.15 d^−1^). The propionate- and acetate-oxidizing bacteria in the two methanogenic chemostats were investigated combining DNA-stable isotope probing (DNA-SIP) and 16S rRNA gene high-throughput sequencing. The results of DNA-SIP with ^13^C-propionate/acetate suggested that, *Smithella*, *Syntrophobacter*, *Cryptanaerobacter*, and unclassified *Rhodospirillaceae* may be putative propionate-oxidizing bacteria; unclassified *Spirochaetaceae*, unclassified *Synergistaceae*, unclassified *Elusimicrobia*, *Mesotoga*, and *Gracilibacter* may contribute to acetate oxidation; unclassified *Syntrophaceae* and *Syntrophomonas* may be butyrate oxidizers. By DNA-SIP, unclassified OTUs with 16S rRNA gene abundance higher than 62% of total *Bacteria* in the PL chemostat and 38% in the PH chemostat were revealed to be related to the degradation of propionate. These results suggest that a variety of uncultured bacteria contribute to propionate degradation during anaerobic digestion. The functions and metabolic characteristics of these bacteria require further investigation.

## Introduction

Anaerobic digestion is a key technology in the development of a future bio-based economy because of the mineralization of organic pollutants in waste/wastewater and simultaneous production of methane as clean energy. Converting organic matter into biogas relies upon an intricate balance of multiple trophic microbial groups, including hydrolytic and fermentative bacteria, syntrophic acetogenic bacteria, and methanogenic archaea^1^. Volatile fatty acids (VFAs) are the major intermediate metabolites during anaerobic digestion. In thermodynamics, propionate is more difficult to anaerobically oxidize than other VFAs such as butyrate and valerate. Propionate oxidation is energetically unfavorable with a standard change in Gibbs free energy (ΔG°) of +76 kJ per mol reaction. The propionate oxidation under methanogenic condition can be realized only by syntrophic association of propionate-oxidizing bacteria and hydrogenotrophic methanogens. Therefore, propionate often accumulates when anaerobic digestion becomes unstable^2^. The oxidation of intermediate propionate to acetate and hydrogen is regarded as the rate-limiting step in anaerobic digestion.

Owing to their symbiotic relationships with methanogens, isolating syntrophic propionate-oxidizing bacteria (SPOB) is challenging. Until now, only eleven SPOB species affiliated with the genera *Syntrophobacter*, *Desulfotomaculum*, *Pelotomaculum*, *Smithella*, and candidatus *Desulfonatronobulbus* have been isolated^3–12^, and a SPOB named *Candidatus* Syntrophosphaera thermopropionivorans is identified in a thermophilic propionate-degrading enriched culture^13^. The limited knowledge of SPOB leads to a gap in knowledge of syntrophic propionate degradation during anaerobic digestion. DNA-based molecular techniques permit the comprehensive determination of microbial diversity but do not generally reveal the relationship between the taxonomy and function of microorganisms. Stable isotope probing (SIP) provides a link between phylogeny and function, and in recent years, this method has been widely used to identify the functions of uncultured microorganisms in various environments^14,15^. Several reports demonstrated that SIP is an effective tool for identifying potential organic acid-degraders in different methanogenic habitats^14,16^. Species other than *Syntrophomonas* have been found to be responsible for syntrophic butyrate-oxidation, such as the genera *Tepidanaerobacter*, *Clostridium*, the families *Syntrophospora*, *Syntrophomonadaceae*, *Syntrophaceae* and the phylum *Actinobacteria*^17^. Some species related to genera *Thermacetogenium* and unclassified *Thermoanaerobacteraceae* were reported responsible for syntrophic acetate oxidation^18^. In addition to the known SPOB genera *Syntrophobacter*, *Pelotomaculum*, and *Smithella*, *Geobacter* and some other anaerobic bacteria affiliated with family *Rhodocyclaceae*, class *Thermomicrobia*, and phyla *Acidobacteria* and *Actinobacteria* were labeled by ^13^C_3_-propionate, which might utilize propionate or metabolic intermediates of propionate oxidation^14^. To the best of our knowledge, no study investigated the SPOB in methanogenic reactor by DNA-SIP up to now. To link the uncultured bacteria in methanogenic reactors with the propionate degradation would be essential for revealing the microbial mechanism of propionate degradation and therefore would contribute to the regulation of anaerobic digestion processes.

In anaerobic reactors treating actual waste/wastewaters, microbial communities are very complex and SPOB abundance is generally low. Therefore, it is difficult to target SPOB using ^13^C_3_-propionate. In this study, we established two mesophilic methanogenic chemostats fed with propionate as the sole carbon source. Stable propionate-degrading microbial communities were obtained through long-term cultivation and enrichment, which is conducive to the effective identification of propionate- and acetate-oxidizing bacteria. Considering that dilution rate (the reciprocal of hydraulic retention time) could seriously affect community structure^19^, two chemostats were operated at different dilution rates (0.05 and 0.15 d^−1^). The potential SPOB in these two chemostats were investigated using DNA-SIP combined 16S rRNA gene high-throughput sequencing. Acetate-oxidizing bacteria were investigated simultaneously, as acetate was the intermediate metabolite of propionate oxidation.

## Results

### Microbial communities in propionate-fed chemostats operated at different dilution rates

The operation performance of each chemostat is displayed in Fig. S1. Both PL (0.05 d^−1^) and PH (0.15 d^−1^) chemostats maintained steady states during the 1000-day and 130-day operation periods, respectively. The biogas production of the PL and PH chemostat was stable at 300 and 1800 mL L^−1^ d^−1^ during this period, respectively. The methane content in biogas was 65–68% for the PL chemostat and 64–66% for the PH chemostat. The organic acid concentration was below 100 mg L^−1^ in both chemostats. The average VSS concentration was 0.9 g L^−1^ for the PL chemostat and 1.0 g L^−1^ for the PH chemostat. The sludge from each chemostat was used to analyze the respective microbial communities.

**Figure 1:**
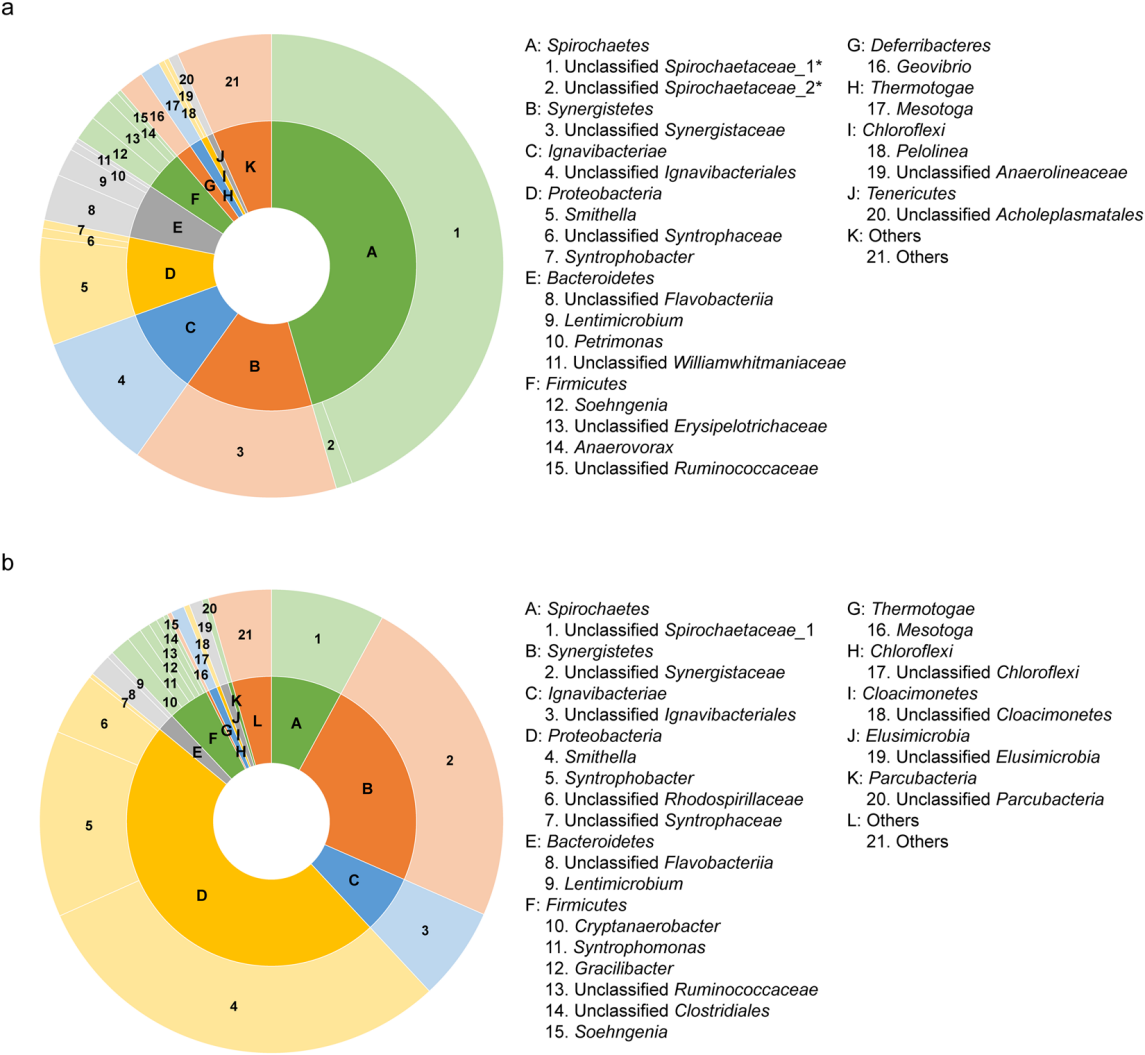
Composition of bacterial community in two mesophilic propionate-fed chemostats. (**a**) PL chemostat: operated at a dilution of 0.05 d^−1^; (**b**) PH chemostat: operated at a dilution of 0.15 d^−1^. *Unclassified *Spirochaetaceae*_1 and unclassified *Spirochaetaceae*_2* were two different genera affiliated to the family *Spirochaetaceae*.

The ratio of bacteria to archaea in the PL and PH chemostats was 0.89 and 1.21, respectively. The bacterial community structure in each chemostat was obviously distinct (Fig. 1). The total abundance of the top 20 bacterial genera accounted for 93.36% and 95.59% of total bacteria in PL and PH, respectively (Fig. 1). Of the bacteria in the PL chemostat, 88.70% were assigned to six phyla: *Spirochaetes* (45.49%), *Synergistetes* (14.34%), *Ignavibacteriae* (9.64%), *Proteobacteria* (8.72%), *Bacteroidetes* (6.07%), and *Firmicutes* (4.44%) (Fig. 1a). Of the bacteria in the PH chemostat, most were assigned to the phylum *Proteobacteria* (47.90%), followed by the phyla *Synergistetes* (23.64%), *Spirochaetes* (7.93%), *Ignavibacteriae* (6.50%), *Firmicutes* (4.61%), and *Bacteroidetes* (1.98%) (Fig. 1b). Unclassified *Spirochaetaceae*_1, unclassified *Synergistaceae*, unclassified *Ignavibacteriales*, and *Smithella* were the dominant genera at the dilution rate of 0.05 d^−1^ (PL chemostat), accounting for 44.34%, 14.34%, 9.64%, and 7.48% of the total bacterial reads, respectively. These genera were also detected in the PH chemostat, but their levels of abundance differed. Among them, *Smithella* is known as SPOB^4^. It was the predominant genus in the PH chemostat and accounted for 30.23%. The other pure cultured SPOB genus *Syntrophobacter*^3^ was also detected in both chemostats, and accounted for 0.59% and 13.00% in PL and PH, respectively. It is worth noting that 76.60% and 48.13% of total bacteria in PL and PH, respectively, was unable to be categorized to known genera, suggesting that a variety of unknown bacteria propagate in propionate-degrading bacterial communities.

The archaeal community of the two chemostats was listed in Table S1. In PL, hydrogenotrophic *Methanoculleus* (92.72%) and acetoclastic *Methanothrix* (6.18%) were the primary genera. In PH, *Methanothrix* (45.58%) became the most dominant methanogen, followed by *Methanospirillum* (26.92%), *Methanoculleus* (17.65%), and *Methanolinea* (7.72%). The abundance of acetoclastic methanogens and the diversity of hydrogenotrophic methanogens were higher than that of PL chemostat.

### DNA-SIP analysis

**Figure 2:**
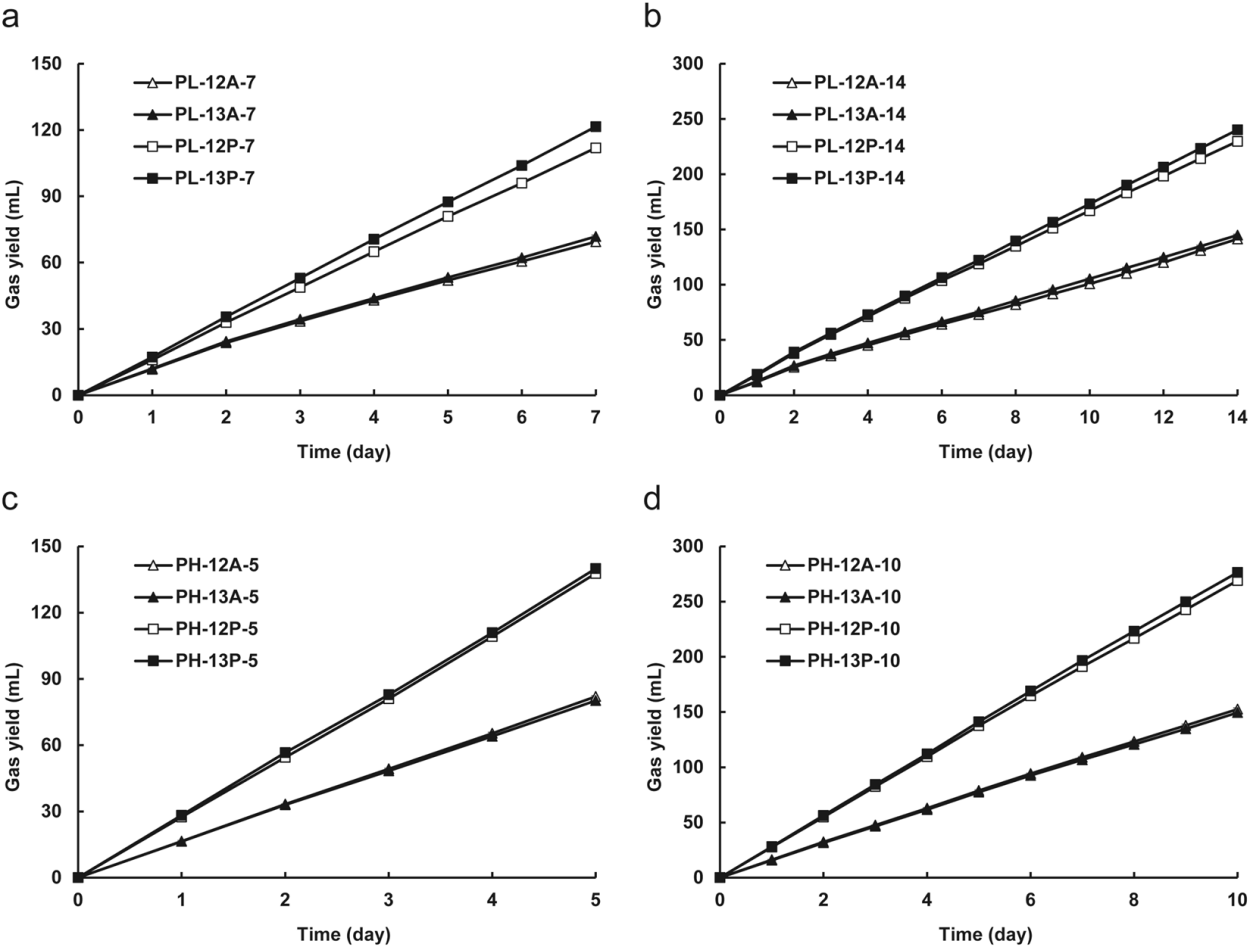
Biogas yields of DNA-SIP incubations with PL (**a**,**b**) and PH (**c**,**d**) sludge. Error bars represent the standard deviation of three replicates.

Sludges from the PL and PH chemostats were incubated with ^13^C- and ^12^C-substrates for 7 d/14 d and 5 d/10 d, respectively (Table 1). The VFAs consumption and biogas yield in each microcosm were measured. Three biological replicates of each treatment exhibited almost the same biogas yield (Fig. 2) and VFA consumption. The sludge obtained from the PL chemostat consumed over 96% propionate and acetate, and the biogas yields were approximately 62–72% of the theoretical value. For the PH chemostat-derived sludge, over 94% added propionate and acetate were consumed, and the biogas yields were approximately 70–87% of the theoretical value.

**Table 1:** Set up of DNA-SIP incubation.

Treatment	Inoculum	Substrate (concentration)^a^	Incubation time
PL-12P-7	PL sludge	^12^C_3_-propionate (12 mM)	7 d
PL-13P-7	PL sludge	^13^C_3_-propionate (12 mM)	7 d
PL-12A-7	PL sludge	^12^C_2_-acetate (12 mM)	7 d
PL-13A-7	PL sludge	^13^C_2_-acetate (12 mM)	7 d
PL-12P-14	PL sludge	^12^C_3_-propionate (12 mM)	14 d
PL-13P-14	PL sludge	^13^C_3_-propionate (12 mM)	14 d
PL-12A-14	PL sludge	^12^C_2_-acetate (12 mM)	14 d
PL-13A-14	PL sludge	^13^C_2_-acetate (12 mM)	14 d
PH-12P-5	PH sludge	^12^C_3_-propionate (12 mM)	5 d
PH-13P-5	PH sludge	^13^C_3_-propionate (12 mM)	5 d
PH-12A-5	PH sludge	^12^C_2_-acetate (12 mM)	5 d
PH-13A-5	PH sludge	^13^C_2_-acetate (12 mM)	5 d
PH-12P-10	PH sludge	^12^C_3_-propionate (12 mM)	10 d
PH-13P-10	PH sludge	^13^C_3_-propionate (12 mM)	10 d
PH-12A-10	PH sludge	^12^C_2_-acetate (12 mM)	10 d
PH-13A-10	PH sludge	^13^C_2_-acetate (12 mM)	10 d

^a^Substrate was added every day, and the substrate concentration was the final concentration of each time addition.

**Figure 3:**
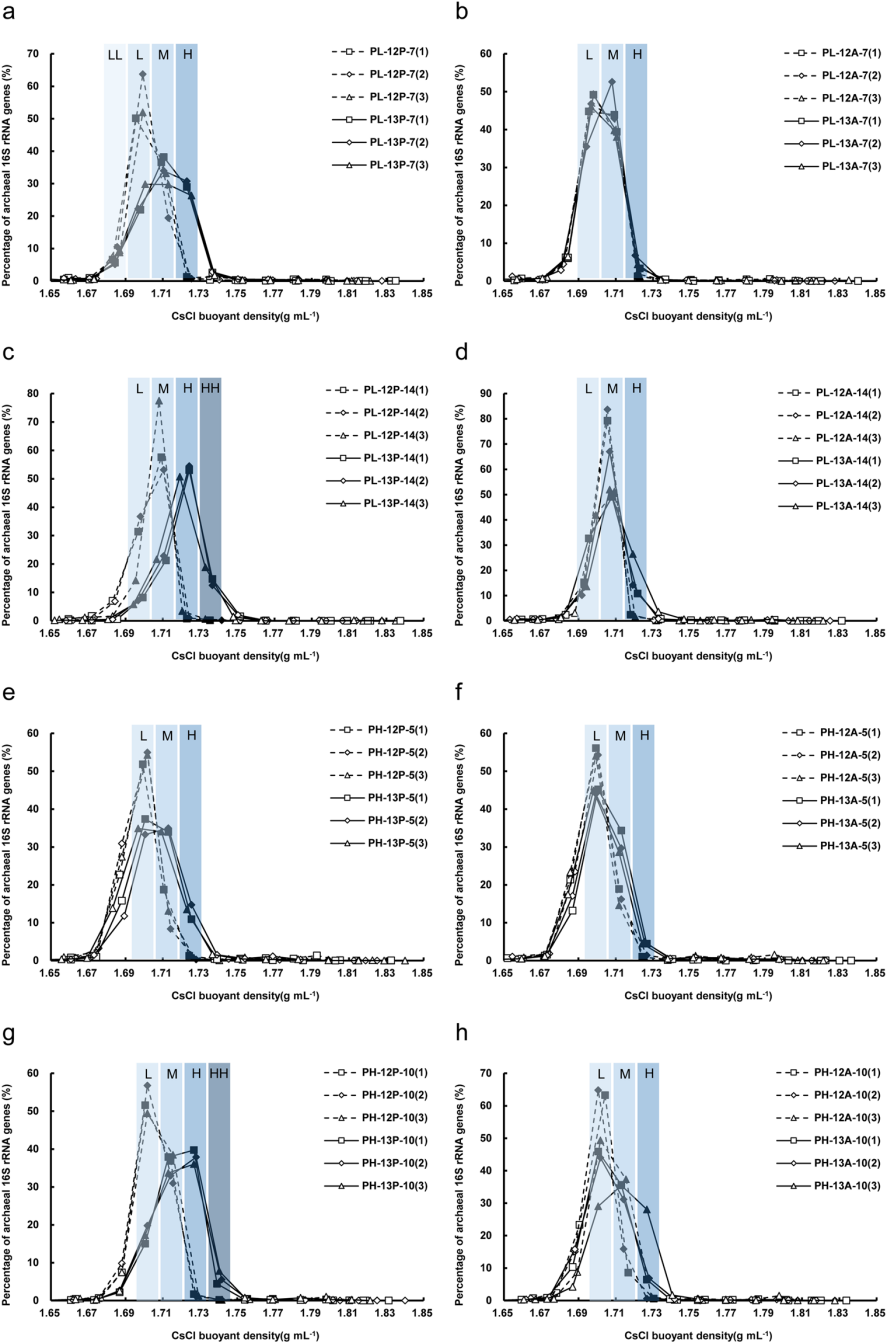
Relative abundance of bacterial 16S rRNA genes in the gradient fractions. (**a**) PL sludge (0.05 d^−1^), propionate treatments, 7 d-incubation; (**b**) PL sludge, acetate treatments, 7 d-incubation; (**c**) PL sludge, propionate treatments, 14 d-incubation; (**d**) PL sludge, acetate treatments, 14 d-incubation; (**e**) PH sludge (0.15 d^−1^), propionate treatments, 5 d-incubation; (**f**) PH sludge, acetate treatments, 5 d-incubation; (**g**) PH sludge, propionate treatments, 10 d-incubation; (**h**) PH sludge, acetate treatments, 10 d-incubation. (1), (2) and (3) in each figure means three replicates. The fractions labeled with filled dot were used for 16S rRNA gene sequencing. The filled dot in (**a**) named LL, L, M and H from buoyant density 1.68–1.73 g mL^−1^; the filled dot in (**b**,**d**,**e**, **f**,**h**) named L, M and H from buoyant density 1.69–1.73 g mL^−1^; the filled dot in (**c**,**g**) named L, M, H and HH from buoyant density 1.69–1.74 g mL^−1^.

DNA samples from each microcosm were used for density-gradient centrifugation and fractionation. The DNA distribution profiles in different density fractions were illustrated using relative copies of bacterial and archaeal 16S rRNA genes (Figs 3 and S2). DNA from ^12^C-substrate treatments was mainly distributed between buoyant density values of 1.68–1.72 g mL^−1^. However, the peak DNA moved to a heavier buoyant density for DNA obtained from ^13^C-substrate treatments. As shown in Fig. 3a, the heavy density fractions from 1.72 to 1.73 g mL^−1^ (fraction H) contained more bacterial 16S rRNA genes in the ^13^C_3_-propionate treatments (10.81–13.83%) than that in the ^12^C_3_-propionate treatments (0.59–0.90%) with PL sludge after a 7-day incubation period. Fig. S2a showed that the abundance of archaeal 16S rRNA genes increased from 0.75–1.28% (^12^C_3_-propionate treatments) to 26.34–30.76% (^13^C_3_-propionate treatments) in the heavy density fractions. For the ^13^C_2_-acetate treatments with PL sludge after a 7-day incubation period, no obvious enriched DNA was found in heavy fractions (Figs 3b and S2b). After the 14-day incubation period, bacterial and archaeal 16S rRNA genes were enriched more significantly in heavy fractions of ^13^C-substrate treatments compared with those after the 7-day incubation period (Figs 3c,d and S2c,d). Particularly, 12.47–18.84% bacterial 16S rRNA genes were enriched at the density fractions from 1.73 to 1.74 g mL^−1^ (fraction HH) in the ^13^C_3_-propionate treatments (Fig. 3c).

For the ^13^C_3_-propionate treatments with PH sludge, a small amount DNA (4.47–7.64%) was enriched in heavy fractions (1.72–1.73 g mL^−1^) after a 5-day incubation period (Fig. 3e). The abundance of bacterial 16S rRNA genes increased from 10.88–16.19% (^12^C_2_-acetate treatments) to 18.78–23.47% in middle fractions (1.71–1.72 g mL^−1^) for the ^13^C_2_-acetate treatments (Fig. 3f). Archaeal 16S rRNA genes were enriched in heavy fractions for ^13^C_3_-propionate and ^13^C_2_-acetate treatments (Fig. S2e,f). After 10-d incubation, both bacterial and archaeal 16S rRNA genes were enriched in heavy fractions (1.72–1.73 mg L^−1^) for ^13^C_3_-propionate and ^13^C_2_-acetate treatments, and 4.43–7.81% bacterial 16S rRNA genes were enriched at the density fractions from 1.73 to 1.74 g mL^−1^ (fraction HH) in the ^13^C_3_-propionate treatments (Fig. 3g). These results suggest that bacterial and archaeal 16S rRNA genes were labeled successfully by ^13^C-acetate and propionate. In order to more closely reflect potential propionate/acetate-oxidizing bacteria and cooperators, recovered DNA of the fractions labeled with filled dots in Fig. 3 and 2S were used for high-throughput sequencing of the 16S rRNA gene.

### Phylogenetic identification of the labeled bacterial species

**Figure 4:**
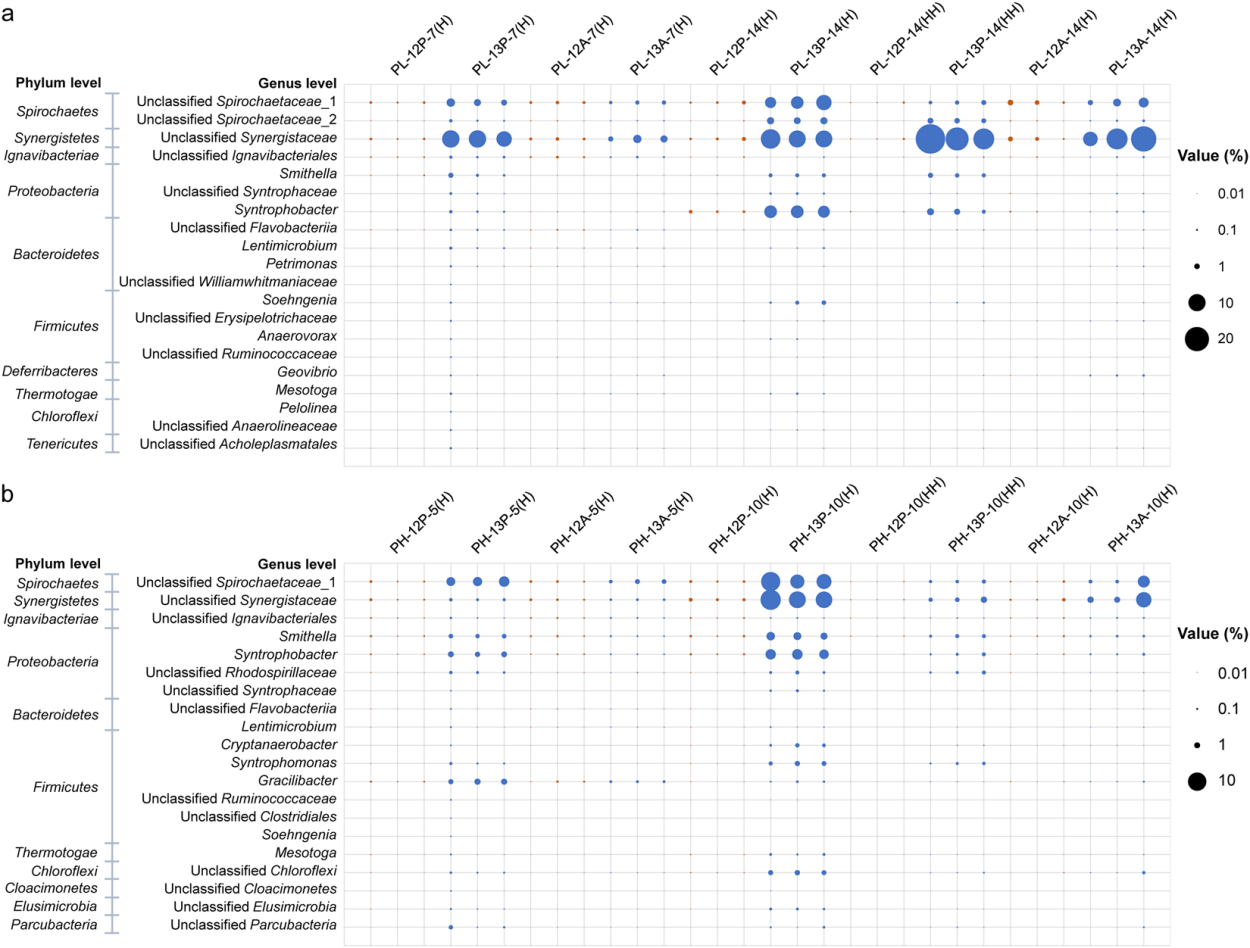
Relative abundance of main bacterial genera (top20 in chemostat) in the heavy fractions of ^13^C- and ^12^C-substrate treatments. (**a**) PL sludge; (**b**) PH sludge.

Bacterial community distribution in different buoyant density fractions is shown in Figs S3–S6. For ^12^C-treatments, the amount of bacterial 16S rRNA genes in each fraction of the same treatment was vastly different, but the structure of bacterial community was similar. For ^13^C-treatments, the structure of the bacterial community in each fraction of the same treatment exhibited distinct differences, which suggested that ^13^C-substrate enriched some species in the heavy fractions. After a 7-day incubation period with PL sludge, in fraction H of ^13^C_3_-propionate treatments and ^13^C_2_-acetate treatments, the abundances of bacterial 16S rRNA genes increased 15.8-fold and 2.2-fold, respectively, compared with the ^12^C-treatment controls (Fig. S3e). Unclassified *Synergistaceae* increased from 0.17% in ^12^C_3_-propionate treatments to 9.06% in ^13^C_3_-propionate treatments (53.6-fold increase) (Fig. 4a). Unclassified *Spirochaetaceae*_1 and unclassified *Spirochaetaceae*_2 increased 8.1- and 6.2-fold, respectively. The two known SPOB *Smithella* and *Syntrophobacter* also increased 7.0- and 13.2-fold, respectively. For the ^13^C_2_-acetate treatments with PL sludge, only unclassified *Synergistaceae* was obviously enriched in fraction H suggesting its role in acetate oxidation, and its abundance increased from 0.30% in ^12^C_2_-acetate treatments to 1.56% in ^13^C_2_-acetate treatments. The low enrichment of unclassified *Synergistaceae* in ^13^C_2_-acetate treatments suggested that high concentrations of acetate in acetate treatments was more easily utilized directly by acetoclastic methanogens. Unclassified *Synergistaceae* might have higher acetate affinity and metabolic activity under low acetate concentrations in propionate treatments. After a 14-day incubation period, bacterial 16S rRNA genes were enriched more obviously in ^13^C_3_-propionate treatments, and reached 25.83% and 25.10% in fraction H and HH, which was 25.5- and 104.2-fold of that in ^12^C_3_-propionate treatments, respectively (Fig. S4e). In addition to the five genera labeled by ^13^C_3_-propionate after a 7-day incubation period, three more genera (*Soehngenia*, *Mesotoga*, and unclassified *Syntrophaceae*) were enriched in fraction H (Fig. 4a). The abundance of them increased 49.6-, 32.6-, and 13.2-fold more than those in ^12^C_3_-propionate treatments. In fraction HH, the labeled genera were similar with ^13^C_3_-propionate treatments after 7-day incubation period. Unclassified *Synergistaceae* was still the predominant bacteria in ^13^C_3_-propionate treatments, and the abundance increased 408.3-fold more than that in ^12^C_3_-propionate treatments. For ^13^C_2_-acetate treatments, the abundance of bacterial 16S rRNA genes in fraction H after a 14-day incubation period was much higher than that after a 7-day incubation period, which suggested that more bacteria were labeled by ^13^C_2_-acetate. However, only unclassified *Synergistaceae* was notably enriched (25.3-fold) in fraction H, which was consistent with the result after the 7-day incubation period. In addition, unclassified *Spirochaetaceae*_1 was lightly enriched (2.4-fold) in fraction H of ^13^C_2_-acetate treatments.

After incubating for 5 days, the abundance of bacterial 16S rRNA genes in ^13^C_3_-propionate treatments with PH sludge increased 7.5-fold in fraction H and 1.9-fold in ^13^C_2_-acetate treatments, compared with their ^12^C-controls (Fig. S5e). Compared with ^12^C_3_-propionate treatments, the abundance of the two known SPOB *Smithella* and *Syntrophobacter* increased 5.0-fold and 13.7-fold in the fraction H of ^13^C_3_-propionate treatments, respectively (Fig. 4b). Unclassified *Spirochaetaceae*_1 increased from 0.13% in ^12^C_3_-propionate treatments to 2.42% in ^13^C_3_-propionate treatments (19.2-fold increase in fraction H). In addition, the abundance of unclassified *Rhodospirillaceae*, *Syntrophomonas*, and *Gracilibacter* increased approximately ten-fold in fraction H of the ^13^C_3_-propionate treatments (Fig. 4b). For ^13^C_2_-acetate treatments, the enrichment of the bacterial 16S rRNA gene was not obvious, and only the abundance of unclassified *Spirochaetaceae*_1 and *Gracilibacter* in fraction H were enriched 3.5-fold and 2.5-fold of that in ^12^C_2_-acetate treatments, respectively (Fig. 4b). After a 10-day incubation period, bacterial 16S rRNA genes were enriched more obviously in heavy fractions in ^13^C-substate treatments (Figs 4b and S7). In addition to the six genera labeled by ^13^C_3_-propionate after 5-day incubation period, seven more genera (unclassified *Synergistaceae*, *Cryptanaerobacter*, unclassified *Chloroflexi*, unclassified *Elusimicrobia*, unclassified *Parcubacteria*, *Mesotoga*, and unclassified *Syntrophaceae*) were enriched 7.0~48.1-fold in fraction H by ^13^C_3_-propionate (Fig. 4b). The abundance of bacteria from these genera increased in fraction HH of ^13^C_3_-propionate treatments compared with ^12^C_3_-propionate treatments (Fig. 4b). In addition, unclassified *Synergistaceae* and unclassified *Spirochaetaceae*_1 increased from 0.17% and 0.09% in ^12^C_2_-acetate treatments to 2.92% and 1.29% in ^13^C_2_-acetate treatments in fraction H, respectively (Fig. 4b).

### Phylogenetic identification of labeled archaeal species

The distribution of archaeal community in different buoyant density fractions is presented in Figs S7–S10. For ^13^C_3_-propionate treatments with PL sludge, the abundance of main methanogen genera *Methanoculleus* and *Methanothrix* was observed with significant enrichment in heavy fractions. However, only acetoclastic *Methanothrix* was labeled by ^13^C_2_-acetate. Similar results were found with PH sludge. Main methanogen genera including *Methanothrix*, *Methanospirillum*,*Methanoculleus*, and *Methanolinea* were all labeled by ^13^C_3_-propionate, but only acetoclastic *Methanothrix* was labeled by ^13^C_2_-acetate. These results suggested that an acetate decarboxylation pathway was used for methane formation of the propionate-degrading microbial community when acetate was the sole carbon source.

## Discussion

In this study, two mesophilic anaerobic chemostats fed with propionate as the sole carbon source were constructed and operated at different dilution rates. The potential propionate- and acetate-oxidizing bacteria during anaerobic propionate digestion were investigated using DNA-SIP and 16S rRNA gene high-throughput sequencing. The results indicate that the dilution rate may influence the structure of putative mesophilic propionate- and acetate-oxidizing bacterial communities.

**Figure 5:**
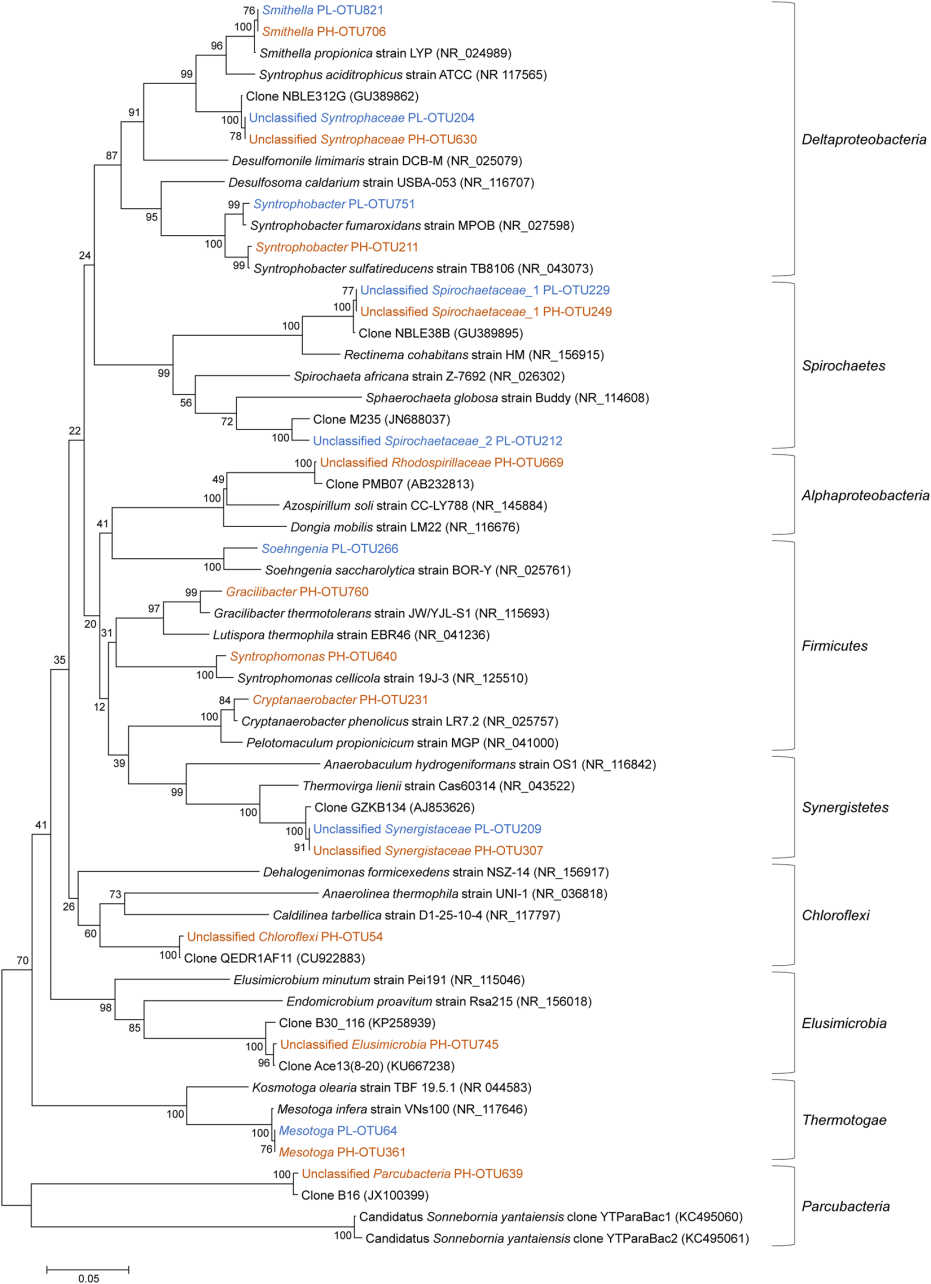
Neighbor-joining phylogenetic tree of ^13^C-substrate labeled representative bacteria.

The phylogenetic relationship of bacterial genera labeled by ^13^C-substrate is shown in Fig. 5. In total, 15 bacterial genera were labeled by ^13^C_3_-propionate or ^13^C_2_-acetate, and 8 of them could not be classified to a genus. These labeled genera exhibited extensive phylogenetic diversity and distributed in 8 phyla (Fig. 5). Four genera (*Smithella*, *Syntrophobacter*, unclassified *Syntrophaceae*, and *Mesotoga*) were labeled by ^13^C_3_-propionate but not ^13^C_2_-acetate with both PL and PH sludge. The label of *Smithella* and *Syntrophobacter*, which are known SPOB, demonstrated the effectiveness of DNA-SIP used in methanogenic community development. *Smithella* and *Syntrophobacter* accounted for 43.23% in PH chemostat, which suggest that they were the primary SPOB under high dilution rate conditions (0.15 d^−1^). The OTUs of unclassified *Syntrophaceae* (PL-OTU204 and PH-OTU630) exhibited a 94% sequence (16S rRNA gene) similarity with SPOB *Smithella propionica* (NR_024989) and a 93% sequence similarity with aromatic compound degrader *Syntrophus aciditrophicus* (NR_117565). Nobu *et al*. reported a novel *Syntrophaceae* member in a methanogenic bioreactor degrading terephthalate (TA) likely performing syntrophic degradation of butyrate and branched-chain fatty acids based on metagenomic and metatranscriptomic analysis^20^, but unclassified *Syntrophaceae* (PL-OTU204 and PH-OTU630) only had a 94% sequence similarity with this novel *Syntrophaceae* member. However, unclassified *Syntrophaceae* (PL-OTU204 and PH-OTU630) were found having a 100% sequence similarity with unclassified *Syntrophaceae* OTUs enriched in two mesophilic isovalerate-fed methanogenic chemostats in our lab, which were verified having butyrate degradation pathway and high expression activity based on metagenomic and metatranscriptomic analysis (unpublished data). Therefore, unclassified *Syntrophaceae* (PL-OTU204 and PH-OTU630) may be related to butyrate oxidation in both PL and PH chemostats. *Syntrophobacter* degrade propionate to produce acetate, hydrogen, and carbon dioxide (propionate + H_2_O → acetate + 3H_2_ + H^+^ + HCO_3_^−^) using the randomizing methyl-malonyl-coenzyme A (CoA) (MMC) pathway, whereas the genus *Smithella* uses a different pathway that results in the formation of acetate and butyrate, followed by the syntrophic *β*-oxidation of butyrate to acetate (2propionate + 2H_2_O → 3acetate + 2H_2_ + H^+^)^21–23^. Unclassified *Syntrophaceae* (PL-OTU204 and PH-OTU630) should be labeled by ^13^C_4_-butyrate, the intermediate metabolite of ^13^C_3_-propionate. *Mesotoga* PL-OTU64 and PH-OTU361 were affiliated to *Mesotoga infera* VNs100 (NR_117646, 99% similarity), which is a member of phylum *Thermotogae*. According to the results of the metagenomic analysis, Nobu *et al*. found that *Mesotoga* may syntrophically oxidize acetate though a previously uncharacterized pathway^20^. However, *Mesotoga* was more easily labeled by ^13^C_3_-propionate than ^13^C_2_-acetate, which suggest that *Mesotoga* may prefer to utilize acetate in very low acetate concentrations.

Unclassified *Spirochaetaceae*_2 and *Soehngenia* were only labeled by ^13^C_3_-propionate with PL sludge suggesting that they might be related to propionate degradation in PL chemostat. Unclassified *Spirochaetaceae*_2 PL-OTU212 exhibited a 98% sequence similarity with clone M235 (JN688037) obtained from an anaerobic cellulolytic microbial consortium^24^. *Soehngenia* PL-OTU266 exhibited 95% sequence similarity with a benzaldehyde-converting bacterium *Soehngenia saccharolytica* (NR_025761)^25^. Six genera (unclassified *Rhodospirillaceae*, *Syntrophomonas*, *Cryptanaerobacter*, unclassified *Chloroflexi*, unclassified *Elusimicrobia*, and unclassified *Parcubacteria*) were labeled by ^13^C_3_-propionate but not ^13^C_2_-acetate with PH sludge. Unclassified *Rhodospirillaceae* PH-OTU669 only exhibited a 91% sequence similarity with a nitrogen-fixing bacterium *Azospirillum soli* (NR_145884), but had a 99% sequence similarity with clone PMB07 (AB232813, 99%) from an enriched mesophilic propionate-degrading methanogenic consortium^19^. Unclassified *Rhodospirillaceae* may be a potential propionate-oxidizing bacterium in the PH chemostat. *Syntrophomonas* PH-OTU640 had a 97% sequence similarity with a syntrophic butyrate-oxidizing bacterium *Syntrophomonas cellicola* (NR_125510)^26^. *Syntrophomonas* should be labeled by ^13^C_4_-butyrate, the intermediate metabolite of ^13^C_3_-propionate. *Cryptanaerobacter* PH-OTU231 exhibited 99% sequence similarity with the *Cryptanaerobacter phenolicus* strain LR7.2 (NR_025757), which can transform phenol and 4-hydroxybenzoate into benzoate^27^. *Cryptanaerobacter* PH-OTU231 also shared a 97% sequence similarity with *Pelotomaculum propionicicum* strain MGP (NR_041000), an obligately syntrophic propionate-oxidizing bacterium^5^. Ahlert *et al*. reported that *Cryptanaerobacter* sp./*Pelotomaculum* sp., whose sequences are related to both genera *Cryptanaerobacter* and *Pelotomaculum*, were the potentially propionate-oxidizing key species^28^. Therefore, *Cryptanaerobacter* PH-OTU231 may participate in syntrophic propionate oxidation. Unclassified *Chloroflexi* PH-OTU54, unclassified *Parcubacteria* PH-OTU639, and unclassified *Elusimicrobia* PH-OTU745 had low sequence similarities (<90%) with pure cultured bacterial species. Unclassified *Chloroflexi* PH-OTU54 exhibited 99% sequence similarity with clone QEDR1AF11 (CU922883) obtained from anaerobic sludge digesters and defined as a core microorganism involved in anaerobic digestion^29^. Nobu *et al*. found an uncultivated *Chloroflexi* subphylum I member which may be capable of H_2_-oxidizing homoacetogenesis in a methanogenic bioreactor degrading terephthalate (TA)^20^. However, unclassified *Chloroflexi* PH-OTU54 only had an 83% sequence similarity with this potential homoacetogen. Unclassified *Parcubacteria* PH-OTU639 was most closely related to clone B16 (JX100399, 99% similarity), which was obtained from an upflow anaerobic sludge blanket reactor with propionate as sole carbon source^30^. PH-OTU54 and PH-OTU639 may be related to propionate oxidation in the PH chemostat as they were labeled only by ^13^C_3_-propionate and no reports on their capacity of acetate oxidation or acetogenesis could be found. Unclassified *Elusimicrobia* PH-OTU745 exhibited 99% sequence similarity with clone Ace13(8–20) (KU667238) labeled by ^13^C_2_-acetate under methanogenic conditions^31^. Considering these findings and the results of the present study, it is suggested that unclassified *Elusimicrobia* PH-OTU745 may be related to the degradation of the intermediate metabolite acetate.

Three genera were labeled by both ^13^C_3_-propionate and ^13^C_2_-acetate, including unclassified *Spirochaetaceae*_1, unclassified *Synergistaceae*, and *Gracilibacter*. The OTUs of unclassified *Spirochaetaceae*_1 (PL-OTU229 and PH-OTU249), predominant bacteria in both PL and PH chemostats, both exhibited 99% similarity with clone NBLE38B (GU389895) from an anaerobic digester treating food-processing wastes^32^. Similar unclassified *Spirochaetaceae* OTUs in an acetate-fed and a butyrate-fed mesophilic methanogenic chemostat were also labeled by ^13^C-acetate^33^. Acetate stimulated the activity of *Spirochaetes* the most compared with other VFAs during anaerobic digestion^34^, and the increase in the cluster II *Spirochaetes* was found to be positively correlated with increase in hydrogenotrophic methanogens in batch reactors seeded with five different anaerobic sludge samples supplemented with acetate as the sole carbon source, suggesting the possible role of *Spirochaetes* in syntrophic acetate oxidation^35^. The OTUs of unclassified *Synergistaceae* (PL-OTU209 and PH-OTU307), predominant bacteria in both PL and PH chemostats, exhibited a 95% similarity with an amino-acid-degrading bacterium *Thermovirga lienii* (NR_043522)^36^. Similar unclassified *Synergistaceae* OTUs in an acetate-fed and a butyrate-fed mesophilic methanogenic chemostat were also labelled by ^13^C-acetate^31,33^. Xu *et al*. reported that *Thermovirga* belonging to *Synergistaceae* accounted for nearly half of the total OTUs in an anaerobic chemostat fed with acetate^37^. In other studies conducted in our lab, a high abundance of this OTU also detected in acetate-fed anaerobic chemostat (data not shown). The syntrophic acetate oxidation ability of *Synergistaceae* spp. were revealed by genome-centric metagenomics^38^ and SIP-based functional study^39^. Together, unclassified *Synergistaceae* (PL-OTU209 and PH-OTU307) may be syntrophic acetate oxidizer in the PL and PH chemostats. *Gracilibacter* PH-OTU760 exhibited 98% sequence similarity with *Gracilibacter thermotolerans* strain JW/YJL-S1 (NR_115693) which can ferment glucose to produce acetate^40^. These species might be able to utilize acetate based on our DNA-SIP results.

Of the archaea, one genus of hydrogenotrophic methanogen *Methanoculleus* was detected at the dilution rate of 0.05 d^−1^, whereas the *Methanoculleus*, *Methanospirillum*, *Methanolinea*, and *Methanobacterium* genera were detected at a higher dilution rate of 0.15 d^−1^. The minimal threshold for hydrogen partial pressures of *Methanoculleus* is much lower than other hydrogenotrophic methanogens^41–43^, which may explain the dominance of *Methanoculleus* among hydrogenotrophic methanogens in the chemostat under the two dilution rates. The higher propionate degradation and hydrogen production rates at a high dilution rate (0.15 d^−1^) allowed the abundance of other hydrogenotrophic methanogens with lower hydrogen affinities to increase. Aceticlastic methanogen *Methanothrix*, which exhibits stronger acetate affinity than *Methanosarcina*^44^, was detected in both chemostats. *Methanothrix* was also found to be the dominant aceticlastic methanogen in a variety of anaerobic reactors at low acetate concentrations^45^, which is consistent with the findings of this study. In the ^13^C_3_-propionate treatments with either PL or PH sludge, all main methanogens in original chemostats were concentrated in the heavy fractions, which suggested that these methanogens participated in propionate-related methanogenesis processes. In the ^13^C_2_-acetate treatments, only the aceticlastic methanogen *Methanothrix* was concentrated in the heavy fractions, which suggest that aceticlastic methanogenesis pathway is dominant in acetate treatments.

In SIP experiment, it is difficult to eliminate cross-feeding, but it is possible to reduce it by optimizing parameters including incubation time, substrate concentration, and substrate adding frequency. Many preliminary SIP experiments had been down to optimize these parameters before the formal SIP experiments carried out in this study. In addition, attention was not payed to those bacteria that were identified in the heavy fraction but having fairly low abundance. Compared with those OTUs having high abundance in the heavy fraction, the bacteria having fairly low abundance might have higher possibility of cross-feeding. However, the functions of the OTUs labeled in this study should be further studied and direct evidences of their functions should be obtained.

In conclusion, DNA-SIP successfully identified the propionate and acetate oxidation players in propionate-fed chemostats. Different species were retrieved from the two chemostats, which were operated at different dilution rates suggesting that, in addition to known SPOB, more uncultured bacteria may be involved in propionate degradation during anaerobic digestion. However, to reveal the propionate and acetate oxidation functions and metabolic pathways of these labeled unknown species, further study employing metagenomics and culture-dependent techniques is required.

## Materials and Methods

### Construction and operation of chemostats fed with propionate as the sole carbon source

Two anaerobic chemostats were constructed using two continuous stirred tank reactors (CSTRs) each with a working volume of 1.8 L as described previously^19^. The seed sludge was obtained from an anaerobic digester treating distillation wastewater of an alcohol plant in Sichuan Province, China. The chemostats were fed with synthetic wastewater containing propionate as the sole carbon source (TOC 8000 mg L^−1^) at dilution rates of 0.05 d^−1^ and 0.15 d^−1^, respectively (designated PL and PH chemostats, respectively). The components of the synthetic wastewater were the same as described in a previous study^19^. The wastewater comprised (grams per liter): sodium propionate, 4.27; propionic acid, 13.16; KH_2_PO_4_, 0.3; KHCO_3_, 4.0; NH_4_Cl, 1.0; NaCl, 0.6; MgCl_2_·6H_2_O, 0.82; CaCl_2_·2H_2_O, 0.08; cysteine–HCl·H_2_O, 0.1; 10 ml of a trace element solution of Deutsche Sammlung von Mikroorganismen und Zellkulturen GmbH medium 318 containing 21.3 mg L^−1^ of NiCl_2_·6H_2_O and 24.7 mg L^−1^ of CoCl_2_·6H_2_O; and 10 ml of vitamin solution of DSMZ medium 318 without B_12_. The two chemostats operated stably at 37 °C for approximately 1500 and 400 days, respectively. During the operation, several parameters including pH, SS, VSS, TOC, and VFAs were measured regularly as described previously^46^. The sludge from each chemostat was used for microbial community analysis and DNA-SIP experiments.

### SIP incubation with ^13^C_3_-propionate and ^13^C_2_-acetate

Sludge was sampled directly from the PL and PH chemostats on day 1467 and 351, respectively. Microcosms were individually constructed using 50-mL serum bottles with 30 mL sludge from PL or PH digester. The headspaces were flushed with pure N_2_ for 3 min, and the bottles were sealed with a butyl rubber stopper and an aluminum cap. Cysteine-HCl was added as reducing agent and resazurin was added as redox indicator. Considering the co-existence of acetate and propionate degraders during the propionate metabolism, the sludge was incubated separately with ^13^C_3_-propionate and ^13^C_2_-acetate to aid in identifying of SPOB. Incubations with ^12^C_3_-propionate and ^12^C_2_-acetate were used as controls. Propionate or acetate were added to the bottles using a gas-tight syringe every day during the incubation period, as shown in Table 1. Sixteen different treatments were established and conducted in triplicate. They are summarized in Table 1. All microcosms were incubated at 37 °C and 150 rpm on a shaker. Biogas production was measured using a syringe every day. After incubation, remaining VFAs were detected and sludge from each microcosm was collected and used for DNA extraction. ^13^C_3_-propionate (99 atom% in ^13^C) and ^13^C_2_-acetate (99 atom% in ^13^C) were purchased from Cambridge Isotope Laboratories (USA).

### DNA extraction, density-gradient centrifugation and fractionation

Total genomic DNA of each sludge sample was extracted by using CTAB method according to the method outlined by Griffiths *et al*.^47^. Purified DNA was prepared for density-gradient centrifugation and fractionation as described by Lueders *et al*.^48^. Briefly, total DNA (~2.5 μg) was added to Quick-Seal polyallomer tubes (6.3 mL, Beckman Coulter, Australia), along with 1.2 mL of the gradient buffer (GB) containing 0.1 M Tris-HCl with pH of 8.0, 0.1 M KCl, and 1 mM EDTA, and 4.8 mL CsCl solution (final buoyant density of 1.90 g mL^−1^). Then, the tubes were sealed, transferred to a Beckman ultracentrifuge with Ti90 fixed angle rotor (Beckman, USA), and centrifuged at 177,000 × g for 40 h at 20 °C. Following centrifugation, 15 density fractions (400 μL of each fraction) were collected from each tube using a fraction recovery system (Beckman Coulter, USA). The buoyant density of each fraction was determined using a digital refractometer (AR200, Reichert, USA)^48^. DNA was recovered from each fraction by PEG6000 precipitation with glycogen.

In order to profile the DNA gradient distribution, bacterial and archaeal 16S rRNA genes in each fraction were quantified by qPCR using the EcoTM real-time PCR system (Illumina, USA) with primer sets Eu27f/Eu518r^49^ and Arch349f/Arch806r^50^, respectively. According to the results of qPCR, the DNA samples from fractions with buoyant density during 1.69–1.74 g mL^−1^ were selected for high-throughput sequencing of the 16S rRNA gene.

### High-throughput sequencing of 16S rRNA gene and phylogenetic analysis

DNA samples were amplified with primer 515 F (5′-GTGCCAGCMGCCGCGGTAA-3′) and 909 R (5′- CCCCGYCAATTCMTTTRAGT -3′) to amplify the V4-V5 region of 16S rRNA gene in both bacteria and archaea. Sequencing was performed with an Illumina MiSeq platform (Illumina, San Diego, USA) according to the standard protocols described by Majorbio Bio-Pharm Technology Co. Ltd. (Shanghai, China). Raw fastq files were quality-filtered by Trimmomatic and merged by FLASH with the following criteria: (i) the reads were truncated at any site receiving an average quality score <20 over a 50 bp sliding window. (ii) Sequences whose overlap exceeded 10 bp were merged according to their overlap with mismatch no more than 2 bp. (iii) Sequences of each sample were separated according to barcodes (exactly matching) and primers (allowing 2 nucleotide mismatching), and reads containing ambiguous bases were removed. Operational taxonomic units (OTUs) were clustered at a 97% similarity cutoff using UPARSE-OTU (version 7.1 http://drive5.com/uparse/) with a novel “greedy” algorithm that simultaneously performs chimera filtering and OTU clustering^51^. Final OTUs were taxonomically classified using Ribosomal Database Project classifiers and NCBI BLAST^52^. Sequences of 21 target OTUs and thirty-seven reference sequences were used to constructed phylogenetic tree. Multiple alignments were run using the Clustalx1.8 (http://www.clustal.org/). Distance matrix trees were constructed using the MEGA4 software package (http://megasoftware.net/mega4/) by using the neighbor-joining method with the Kimura two parameter model and complete-deletion option. Bootstrap resampling analyses for 1000 replicates were performed to estimate the confidence of tree topologies.

### Nucleotide sequence accession numbers

The original sequencing data is available at the National Center for Biotechnology Information (Accession No. PRJNA505222).

## Supplementary information

Supplementary information
