# Supplementary material for: Using DNA-based stable isotope probing to reveal novel propionate- and acetate-oxidizing bacteria in propionate-fed mesophilic anaerobic chemostats
Source: Sci Rep. 2019 Nov 22;9:17396. doi: 10.1038/s41598-019-53849-0 (PMC6874663; doi:10.1038/s41598-019-53849-0)
Supplement: Supplementary file 1 — Supplementary information [file 41598_2019_53849_MOESM1_ESM.pdf]

Supplementary Materials

Using DNA-based stable isotope probing to reveal novel propionate- and acetate-oxidizing bacteria in propionate-fed mesophilic anaerobic chemostats

Authors:

Hui-Zhong Wang[1], Xiao-Meng Lv[2], Yue Yi[1], Dan Zheng[1], Min Gou[1], Yong Nie[3], Bing Hu[3], Masaru K Nobu[4], Takashi Narihiro[4], Yue-Qin Tang[1,*]

Affiliations:

[1]College of Architecture and Environment, Sichuan University, No. 24, South Section 1, First Ring Road, Chengdu, Sichuan 610065, China

[2]Institute of New Energy and Low-Carbon Technology, Sichuan University, No. 24, South Section 1, First Ring Road, Chengdu, Sichuan 610065, China

[3]Department of Energy and Resources, College of Engineering, Peking University, Beijing 100871, China

[4]Bioproduction Research Institute, National Institute of Advanced Industrial Science and Technology (AIST), Tsukuba, 305-8566, Japan

[*]**Corresponding author**

Yue-Qin Tang,

College of Architecture and Environment, Sichuan University, No. 24, South Section 1, First Ring Road, Chengdu, Sichuan 610065, China

Tel: 86-28-85990937; Fax: 86-28-85990936; e-mail: tangyq@scu.edu

Table S1 Archaeal communities in the two mesophilic propionate-fed chemostats*

	PL reactor	PH reactor
Methanothrix	6.18%	45.58%
Methanospirillum	0.00%	26.92%
Methanoculleus	92.72%	17.65%
Methanolinea	0.08%	7.72%
Methanobacterium	0.26%	1.69%
Others	0.76%	0.43%

*PL reactor: operated at a dilution of 0.05 d^{-1}; PH reactor: operated at a dilution of 0.15 d^{-1}

a

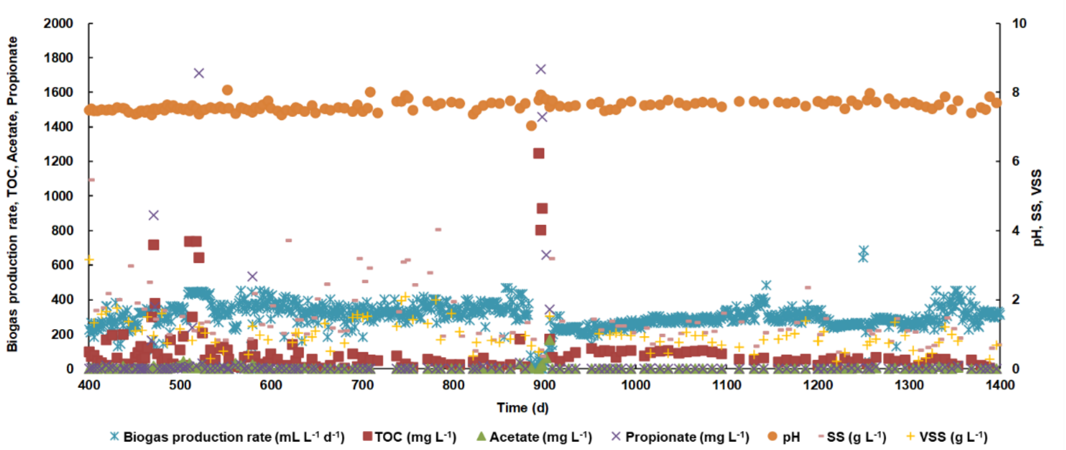

b

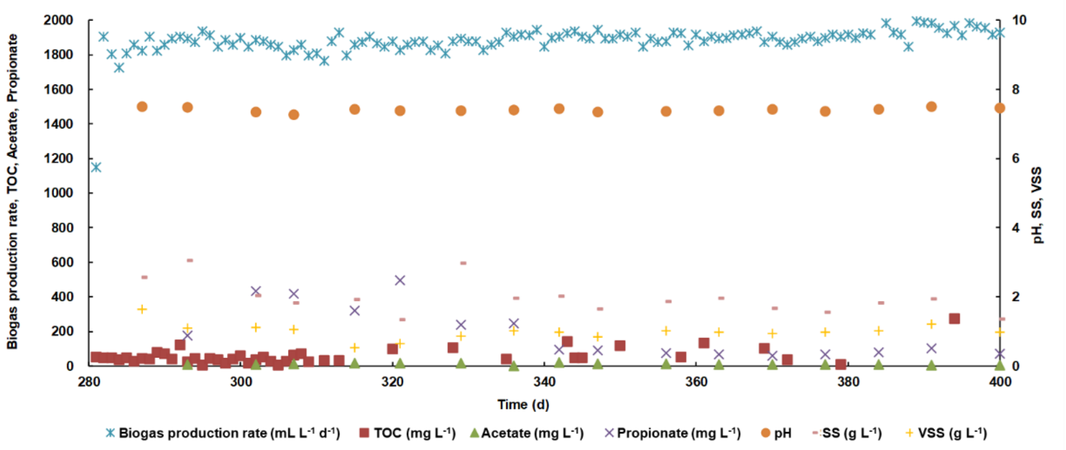

Fig. S1 Performance of the mesophilic chemostats fed with propionate as the sole carbon source. a: PL chemostat (operated at a dilution rate of 0.05 d^{-1}); b: PH chemostat (0.15 d^{-1})

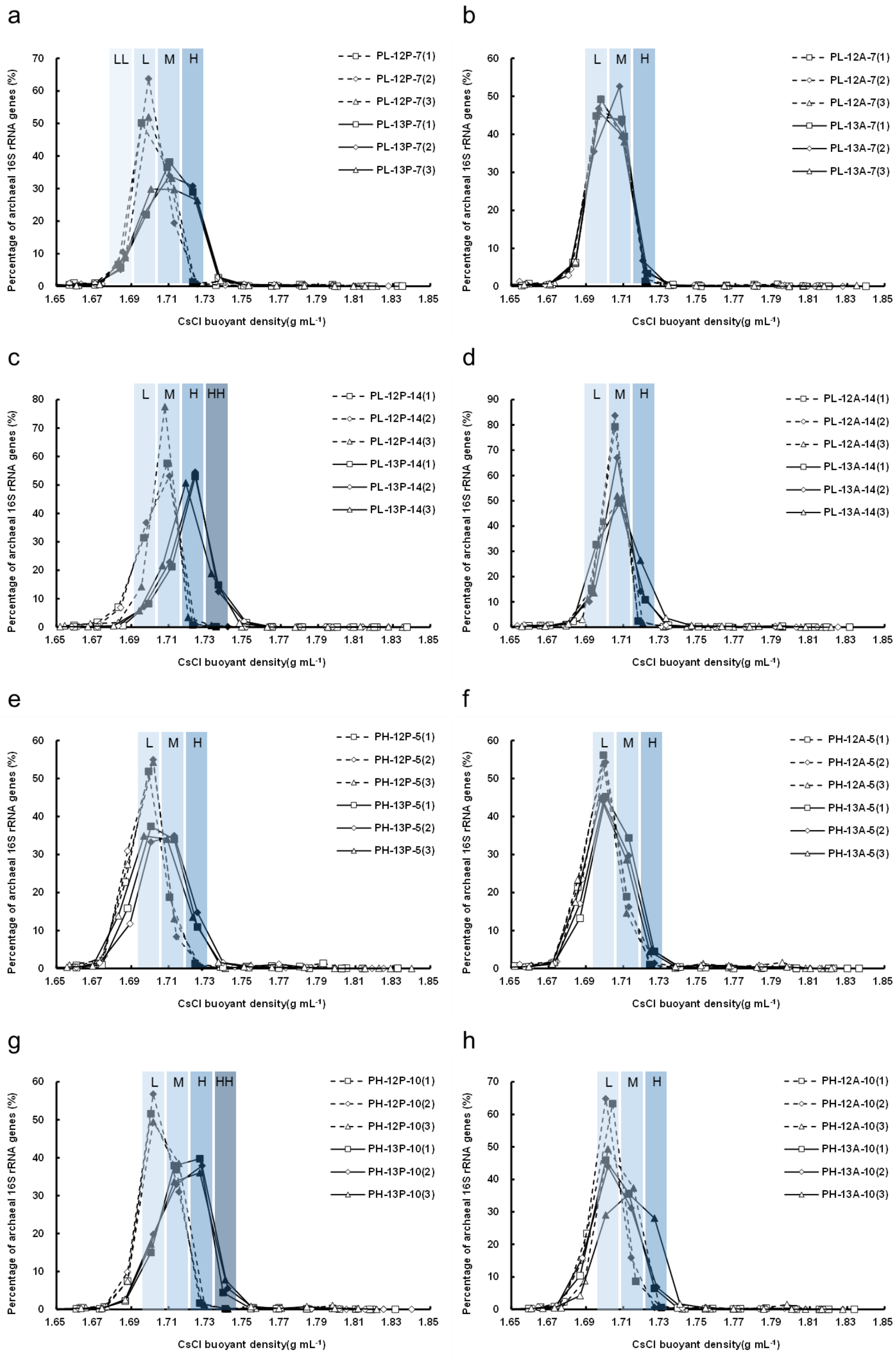

Fig. S2 Relative abundance of archaeal 16S rRNA genes in the gradient fractions. a: PL sludge (0.05 d^{-1}), propionate treatments, 7 d-incubation; b: PL sludge, acetate treatments, 7 d-incubation; c: PL sludge, propionate treatments, 14 d-incubation; d: PL sludge, acetate treatments, 14 d-incubation; e: PH sludge

($0.15\ d^{-1}$), propionate treatments, 5 d-incubation; f: PH sludge, acetate treatments, 5 d-incubation; g: PH sludge, propionate treatments, 10 d-incubation; h: PH sludge, acetate treatments, 10 d-incubation. (1), (2) and (3) in each figure means three replicates. The fractions labeled with filled dot were used for sequencing analysis. The filled dot in (a) named LL, L, M and H from buoyant density 1.68-1.73 g mL^{-1}; the filled dot in (b, d, e, f, h) named L, M and H from buoyant density 1.69-1.73 g mL^{-1}; the filled dot in (c, g) named L, M, H and HH from buoyant density 1.69-1.74 g mL^{-1}

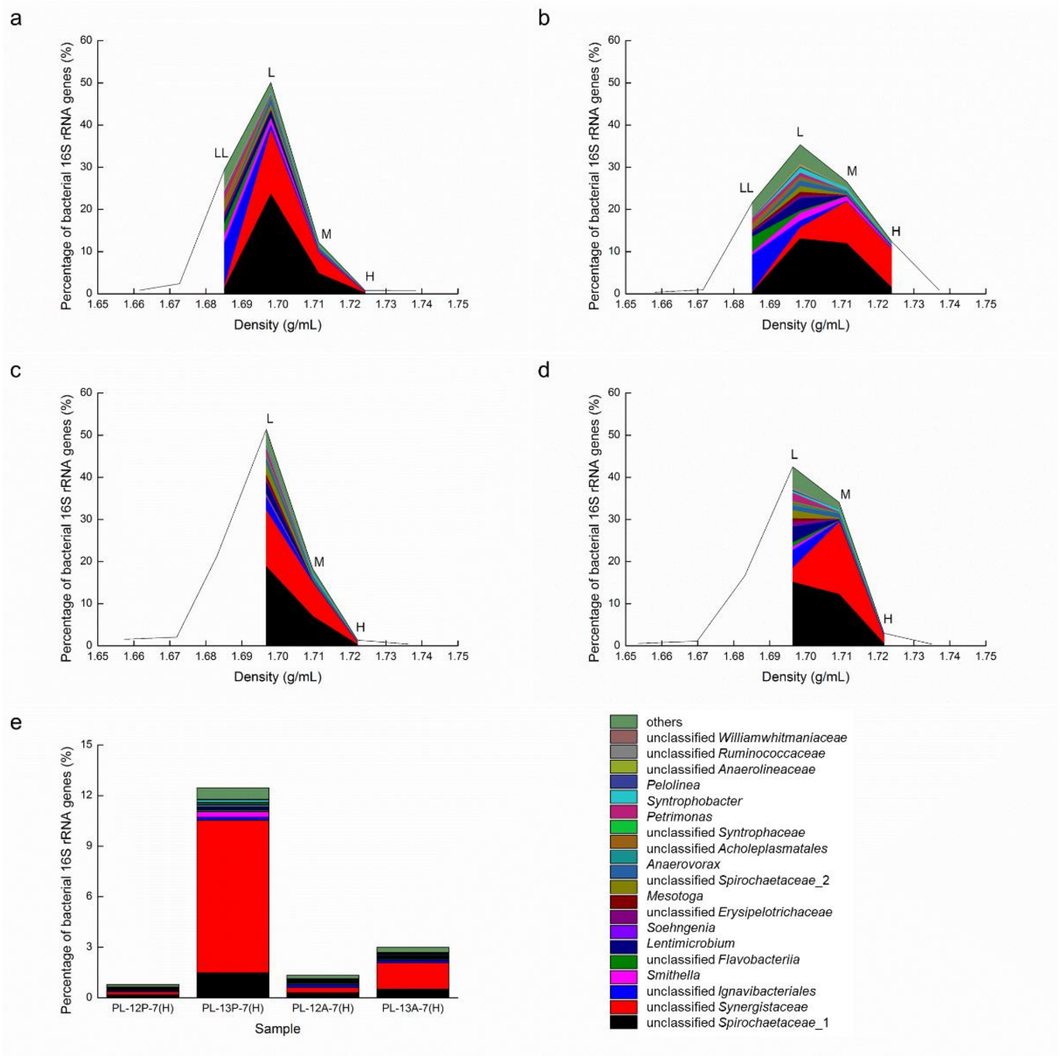

Fig. S3 Relative abundance of bacterial genera in the gradient fractions from PL sludge with 7-d incubation. a: $^{12}C_3$-propionate; b: $^{13}C_3$-propionate; c: $^{12}C_2$-acetate; d: $^{13}C_2$-acetate; e: samples of heavy density fractions

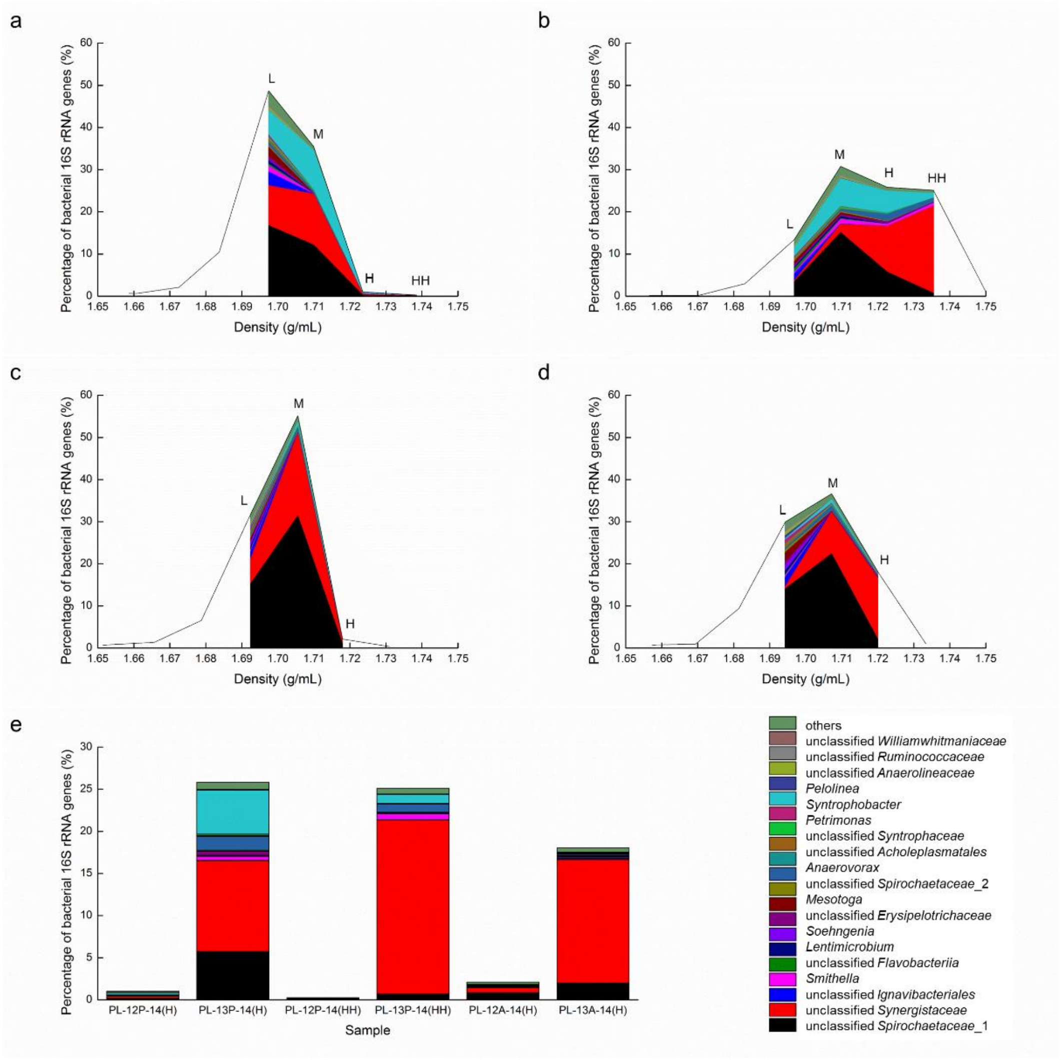

Fig. S4 Relative abundance of bacterial genera in the gradient fractions from PL sludge with 14-d incubation. a: $^{12}C_3$-propionate; b: $^{13}C_3$-propionate; c: $^{12}C_2$-acetate; d: $^{13}C_2$-acetate; e: samples of heavy density fractions

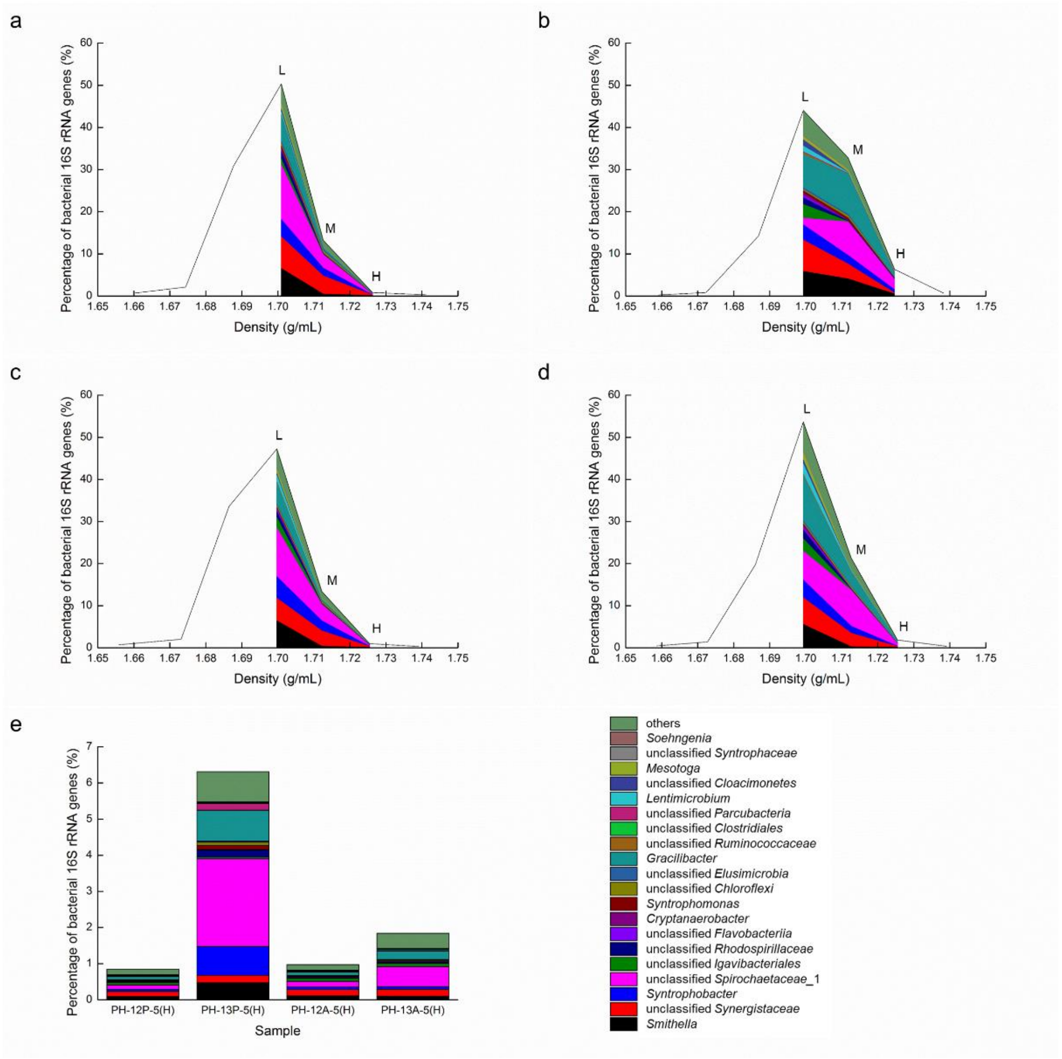

Fig. S5 Relative abundance of bacterial genera in the gradient fractions from PH sludge with 5-d incubation. a: $^{12}C_3$-propionate; b: $^{13}C_3$-propionate; c: $^{12}C_2$-acetate; d: $^{13}C_2$-acetate; e: samples of heavy density fractions

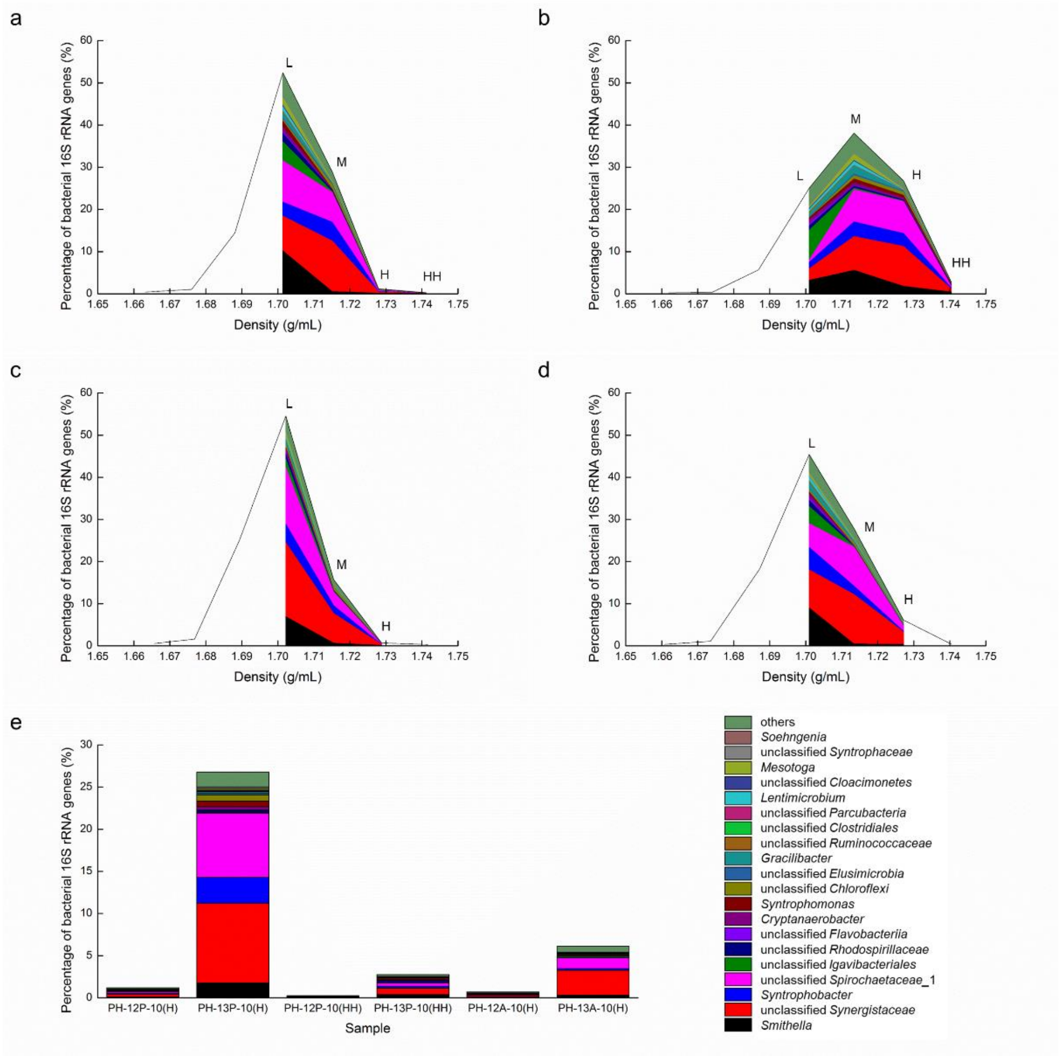

Fig. S6 Relative abundance of bacterial genera in the gradient fractions from PH sludge with 10-d incubation. a: $^{12}C_3$-propionate; b: $^{13}C_3$-propionate; c: $^{12}C_2$-acetate; d: $^{13}C_2$-acetate; e: samples of heavy density fractions

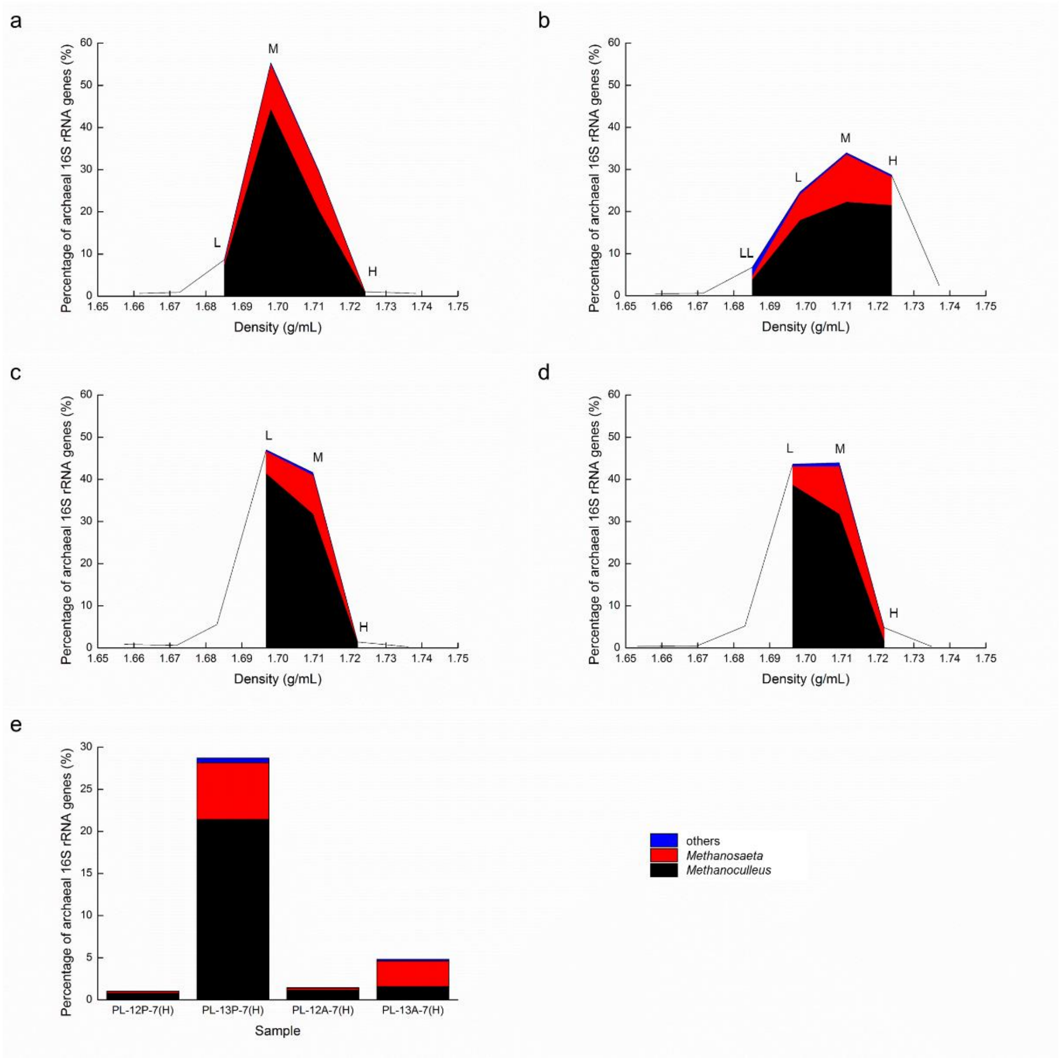

Fig. S7 Relative abundance of archaeal genera in the gradient fractions from PL sludge with 7-d incubation. a: $^{12}C_3$-propionate; b: $^{13}C_3$-propionate; c: $^{12}C_2$-acetate; d: $^{13}C_2$-acetate; e: samples of heavy density fractions

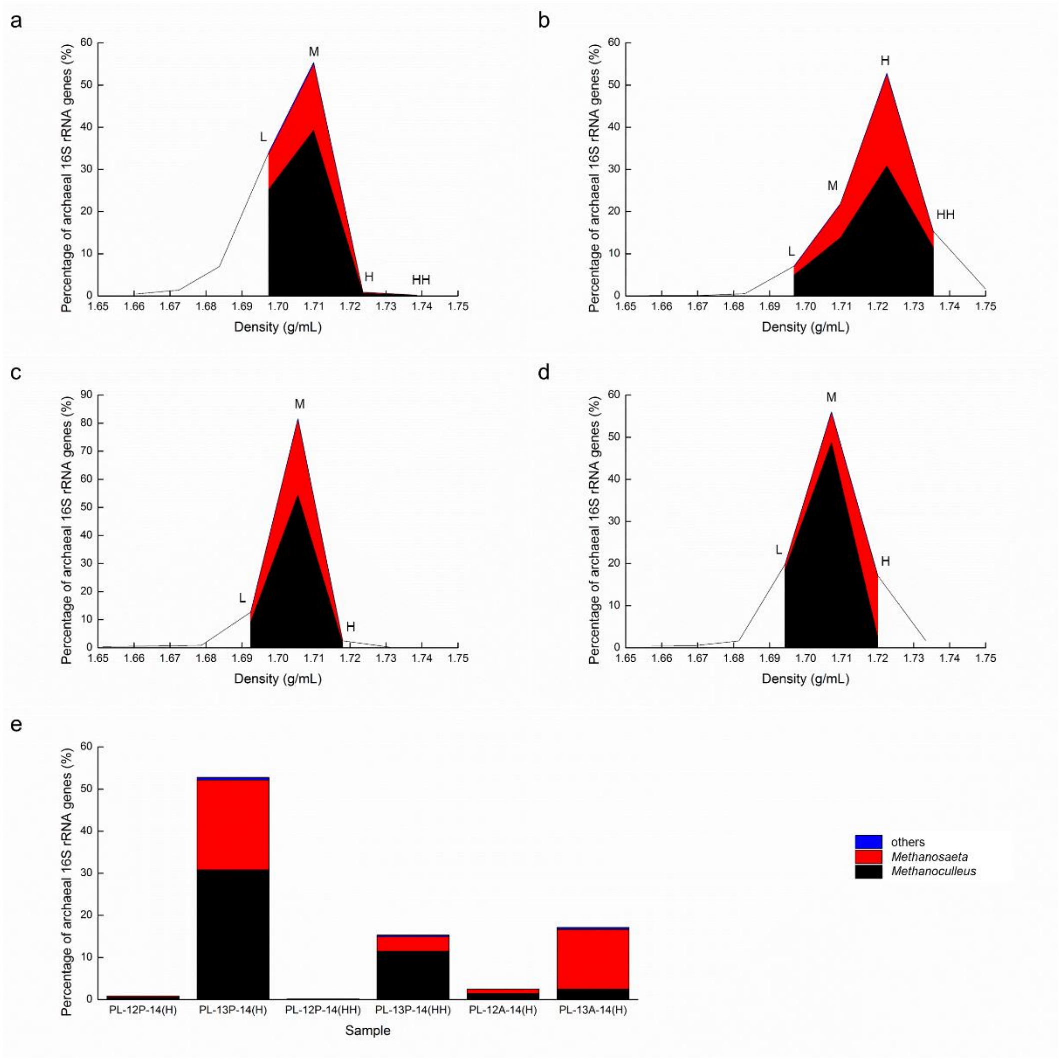

Fig. S8 Relative abundance of archaeal genera in the gradient fractions from PL sludge with 14-d incubation. a: $^{12}C_3$-propionate; b: $^{13}C_3$-propionate; c: $^{12}C_2$-acetate; d: $^{13}C_2$-acetate; e: samples of heavy density fractions

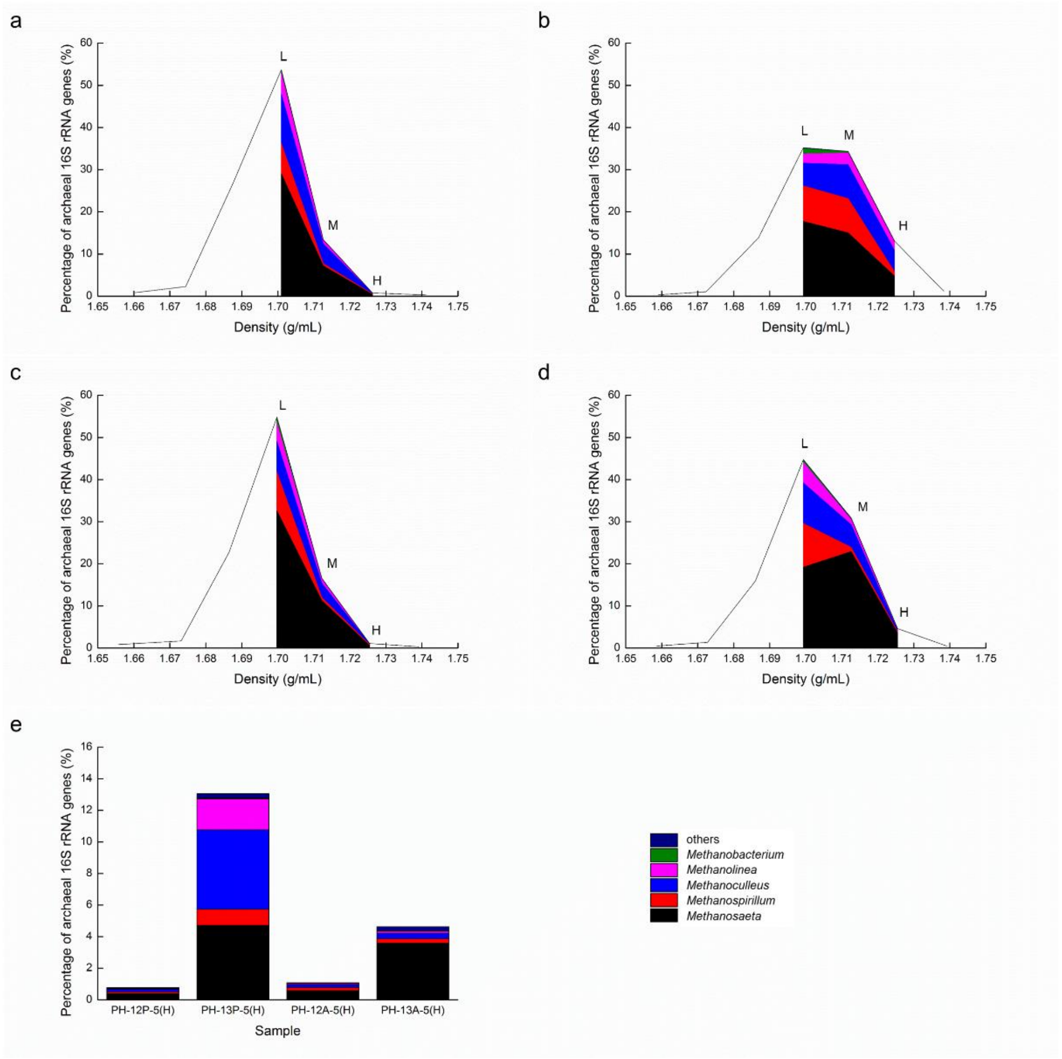

Fig. S9 Relative abundance of archaeal genera in the gradient fractions from PH sludge with 5-d incubation. a: $^{12}C_3$-propionate; b: $^{13}C_3$-propionate; c: $^{12}C_2$-acetate; d: $^{13}C_2$-acetate; e: samples of heavy density fractions

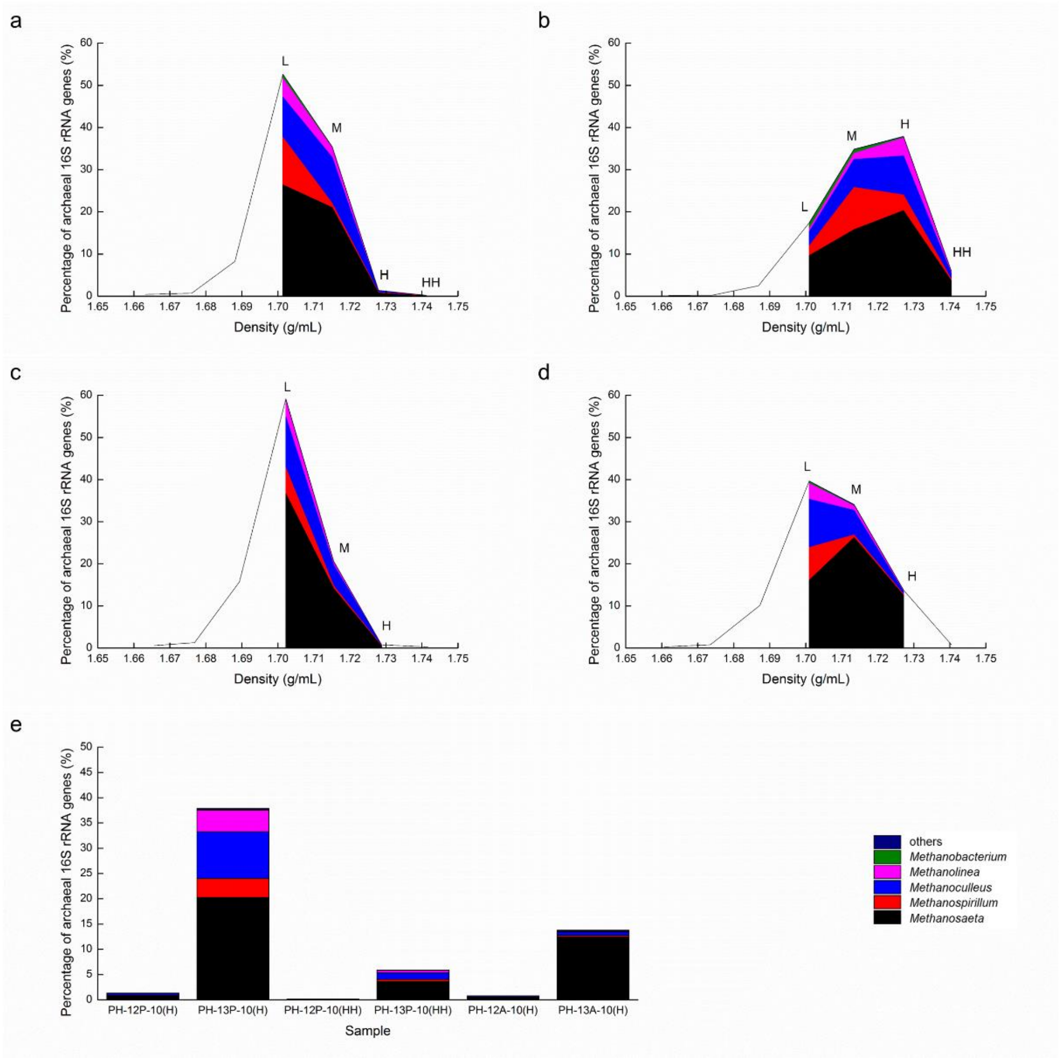

Fig. S10 Relative abundance of archaeal genera in the gradient fractions from PH sludge with 10-d incubation. a: $^{12}C_3$-propionate; b: $^{13}C_3$-propionate; c: $^{12}C_2$-acetate; d: $^{13}C_2$-acetate; e: samples of heavy density fractions
